# Co-Administration of Metformin and N-Acetyl Cysteine Fails to Improve Clinical Manifestations in PCOS Individual Undergoing ICSI

**Published:** 2014-07-08

**Authors:** Ebrahim Cheraghi, Malek Soleimani Mehranjani, Mohammad Ali Shariatzadeh, Mohammad Hossein Nasr Esfahani, Zahra Ebrahimi

**Affiliations:** 1 Medical Sciences Research Laboratory, Department of Biology, Faculty of Science, Arak University, Arak, Iran; 2 Department of Biology, Faculty of Science, Arak University, Arak, Iran; 3 Department of Reproductive Biotechnology at Reproductive Biomedicine Research Center, Royan Institute, ACECR, Isfahan, Iran; 4 Obstetrics and Gynecology Unit, Infertility Treatment Center, ACECR, Qom, Iran

**Keywords:** Polycystic Ovary Syndrome, Metformin, N-Acetyl Cysteine, Insulin Resistance, Oxidative Stress

## Abstract

**Background:**

Studies have demonstrated the efficacy of metformin (MTF ) in reducing insulin resistance and N-acetyl cysteine (NAC) in inhibiting oxidative stress which are involved in the pathogenesis of polycystic ovarian syndrome (PCOS). We aimed to compare the effects of MTF and NAC combination on serum metabolite and hormonal levels during the course of ovulation induction in PCOS individual candidates of intracytoplasmic sperm injection (ICSI).

**Materials and Methods:**

In this prospective randomized clinical trial, placebo con- trolled pilot study, 80 patients of polycystic ovarian syndrome at the age of 25-35 years were divided into 4 groups (n=20): i. NAC=treated with N-acetyl cysteine (600 mg three times daily), ii. MTF=treated with metformin (500 mg three times daily), iii. MTF+NAC=treated with N-acetyl cysteine plus metformin (the offered doses) and iv. placebo (PLA). A total number of 20 patients (6 from MTF group, 4 from NAC group, 6 from MTF+NAC group and 4 from PLA group) were dropped of the study. The drugs were administrated from day 3 of menses of previous cycle until ovum pick-up.

**Results:**

Serum levels of luteinizing hormone (LH), total testosterone, cholester- ol and triglyceride, insulin and leptin significantly reduced in the MTF and NAC groups compared to the placebo (p<0.01). But levels of LH, total testosterone, cholesterol and triglyceride had no significant reduction in the MTF+NAC groups compared to the placebo. The serum levels of malonyldialdehyde (MDA), insulin and leptin reduced significantly after treatment in the MTF+NAC group compared to the placebo (p<0.05).

**Conclusion:**

Considering the adverse effect of combination therapy, we proposed the conadministration might have no beneficial effect for PCOS patient during course of ovulation induction of ICSI (Registration Number: IRCT201204159476N1).

## Introduction

Polycystic ovarian syndrome (PCOS) is an ovarian dysfunction syndrome with the combination of heterogeneous symptoms and signs; it is considered the most prevalent endocrinopathy resulting in anovulation up to 10% of women at reproductive age (1). A rise in the serum levels of androgen, insulin and luteinizing hormone (LH), menstrual dysfunction, hirsutism, infertility and obesity are some of the complications which are associated with PCOS (2). In addition, endometrial hyperplasia, cancer, type II diabetes, hypertension and dyslipidemia are considered as long term consequences of this syndrome (3). Studies have concluded that treatment with insulin-sensitizing agents such as metformin (MTF) results in a decrease of the levels of serum lipids, androgen and insulin; regulation of menstrual cycles; and promotion of both spontaneous and induced ovulation which leads to an increase in pregnancy rate (4). MTF has been used in the treatment of PCOS for a long time; however, gastrointestinal side effects, hypoglycemia and an increase in the serum homocysteine levels in some patients are associated with consuming MTF (5, 6). Hyperhomocysteinaemia is regarded as risk factors for cardiovascular diseases, thrombophilia, pre-eclampsia and recurrent abortion (7, 8).

N-acetyl cysteine (NAC) is an acetylated variant of L-cysteine containing sulfhydryl groups which acts as a powerful antioxidant, antiapoptotic and free-radical scavenger; it also stimulates production of glutathione (9, 10), lowers the serum homocysteine levels and promotes detoxification (11). Since clinical evidence suggests that hyperhomocysteinaemia leads to endothelial dysfunction through generation of reactive oxygen species (12), NAC can preserve vascular integrity and protect against ischemic injuries (13).

Since the effects of co-administration of MTF and NAC compared to MTF and NAC alone in reducing metabolic and hormonal factors such as insulin, leptin and MDA in patients with PCOS has not been evaluated yet, we have evaluated and compared the efficacy of co-treatment of MTF and NAC on clinical, metabolic and hormonal aspects during course of ovulation induction in PCOS individual undergoing ICSI cycle.

## Materials and Methods

### Study population

Through a prospective randomized clinical trial, placebo controlled pilot study, in the interval between July 2012 and February 2013, 80 infertile PCOS individuals at the age of 25-35 years, candidate of ICSI, were enrolled. This study was conducted in the *in vitro* fertilization (IVF) Unit of Infertility Research Center of the ACECR, Qom.

Patients needed to fulfill PCOS diagnostic criteria which was based on the Rotterdam consensus workshop in 2003 (14) as follows: having two out of three criteria of chronic oligo-or anovulation, clinical or biochemical hyperandrogenism, or polycystic ovaries at sonography examination. Hypersensitivity to either MTF or NAC, presence of infertility factors other than anovulation, male infertility, pelvic organic pathologies, congenital adrenal hyperplasia, thyroid dysfunction, Cushing’s syndrome, hyperprolactinemia, androgen secreting neoplasia, diabetes mellitus, consumption of medications affecting carbohydrate metabolism, taking hormonal analogues other than progesterone two months prior to enrollment, and severe hepatic or kidney diseases were considered as exclusion criteria. The semen samples were assessed according to WHO guidelines (15) and individuals with abnormal semen parameters were excluded from study.

An approval from the Research Ethics Committee of Royan Institute, Iran, and an informed consent from participants were obtained. All patients were asked to avoid any changes in their normal physical activity and diet and also not to take any new pharmacotherapy during the study.

### Treatment design

The patients were examined by the gynecologists. The subjects were randomly selected to receive either MTF and NAC or placebo. Eighty patients were divided into four groups (N=20): i. NAC (NAC group) which received NAC (Holzkrichen, Germany, batch no. 6N5483; 600 mg three times daily), ii. MTF group which received MTF (Glucophage, Merck, West Drayton, UK; 500 mg three times daily), iii. MTF+NAC group which were co-treated with MTF and NAC with the offered doses three times daily, and iv. placebo (PLA group) which received oral rehydration salts (ORS, Poursina, Tehran, Iran; batch no.30) three times daily, for a period of six weeks. Before beginning treatment, patients were also asked to report any possible adverse effects during the treatment, and they were evaluated for any presenting main complaints at the end of the treatment period.

A total number of 20 patients (6 from MTF group, 4 from NAC group, 6 from MTF+NAC group and 4 from PLA group) were dropped out due to intolerance of medication, monofollicular development, and failure of ovulation induction during ICSI cycle, and ultimately, 60 patients remained in the study.

### Ovulation induction

Patients with PCOS undergoing ICSI treatment using a long GnRH agonist protocol received MTF, NAC, or MTF+NAC tablets, randomly, from third day of menses of previous cycle until the day of oocyte aspiration.

All patients received oral contraceptive pills (OCPs) for 21 days starting simultaneously with MTF, NAC or MTF+NAC on day 3 of menses of the cycle prior to the treatment cycle. Ovarian down-regulation was initiated with daily buserelin acetate 1 mg (Suprefact, Aventis, Germany), beginning on day 19 of the preceding menstruation (Luteal phase) and after ovarian down-regulation was achieved day 2 of last menstrual period (LMP) and the dose was then reduced to 0.5 mg when the thickness of the endometrium was <4 mm. Ovarian stimulation began with daily injections of average 2 ampoule of recombinant follicle stimulating hormone (rFSH; Gonal-f, Merck Serono S.A., Geneva, Switzerland). Following cycle monitoring, using vaginal sonography (HS 4000, Honda Electronics Co., Japan), ovulation was induced by the administration of average 10,000 IU human chorionic gonadotropin (HCG; Pregnyl, Organon, Netherlands). When at least three follicles had reached diameters of 16-18 mm, transvaginal oocyte aspiration was performed with ultrasound guidance under general anesthesia 36 hours after injection of HCG.

### Assessment of baseline and clinical features

The body mass index (BMI), the waist/hip ratio (WHR) and the blood pressures were recorded for each patient once before the treatment on the third day of menstruation and once in the day of ovum pick up (OPU) of ICSI cycle. Fasting blood samples were collected from each individual once before the treatment on day 3 of menses of previous cycle before ICSI and once during oocyte aspiration. The peripheral blood sample taken from each patient were immediately centrifuged for 10 minutes at 3000 rpm (EBA20, Hettich, UK), and serum samples were stored at -70˚C for future evaluadtion and analysis.

The serum levels of follicle stimulating hormone (FSH, mIU/ml, Cat.N.DE1288), luteinizing hormone (LH, mIU/ml, Cat.N.DE1289), prolactin (PRL, ng/ml, Cat.N.DE1291), total testosterone (TT, ng/ml, Cat.N.DE1559), fasting insulin (mIU/L, Cat.N.DE2935), estradiol (E2, pg/ml, Cat.N.DE2693) and dehydroepiandrosterone sulfate (DHEA-S, ng/ml, Cat.N.DE1562) in all samples were measured using ELISA enzyme immunoassay (Demeditec Diagnostics GmbH, Germany) for hormonal profile. The serum levels of fasting blood sugar (FBS, mg/dl), cholesterol (Chol, mg/dl), high density lipoprotein cholesterol (HDL, mg/dl), low density lipoprotein cholesterol (LDL, mg/dl), triglyceride (TG, mg/dl) and very low density lipoprotein cholesterol (VLDL, mg/dl) were measured using electro-chemical luminescent technique kits (E-411, Roche Company Germany). Serum levels of anti-Mullerian hormone (AMH, ng/ml) were assessed using a second generation enzyme immunoassay (AMH-EIA kit, Cat.N.A92269C, Immunotech Beckman Coulter Company, USA) according to the supplier’s instructions. Serum leptin (ng/ml) level was also measured with the LEPTIN (Human) ELISA Kit (Cat.N.KA0025, Abnova Corporation, Taiwan) using sandwich enzyme immunoassay technique. The level of serum malondialdehyde (MDA, μM), a naturally occurring product of lipid peroxidation, was measured using the thiobarbituric acid (TBA) colorimetric method by TBARS Assay Kit (Cat.N.KA1381, Abnova Corporation, Taiwan).

### Statistical analysis

The normality of continuous variables were confirmed using the Kolmogrov-Smirnov test and data were reported as means ± SD. Data were analyzed with one-way ANOVA and the Tukey’s test for post-hoc analysis. Chi-squared test was used for the statistical analysis where appropriate. Means were considered significantly different at p<0.05. Pearson’s correlation test was performed to define the correlation between variables. Multivariate linear regression analysis was done to test the effect of leptin on hyperinsulinemia and on increased stress oxidative. All data were analyzed with a Statistical Package for the Social Sciences (SPSS Inc., Chicago, IL, USA) version 16.

## Results

### Clinical and demographic characteristics

There were no significant differences in the age, duration of marriage, duration of infertility, the occurrence of oligomenorrhoea, amenorrhoea and hirsutism between the four groups are presented in table 1.

Also, there were no significant differences in weight, height, BMI, waist size, hip size and WHR in the treatment groups are presented in table 2.

**Table 1:** Clinical characteristics of the four groups of PCOS patients


Parameters	Treatment groups with				
	NAC	MTF	NAC+MTF	PlA	P value

**Mean age(25-35 Y)**	29.67±3.35	28.07±3.41	28.67±3.86	27.93 ±2.8	0.491^NS^
**Mean duration of marriage(Y)**	8.07±3.97	8.6±3.2	8.2±2.75	8.57±3.05	0.960^NS^
**Mean duration of infertility(Y)**	6.63±3.63	6.77±3.07	6.3±2.63	6.8±2.95	0.956^NS^
No. of amenorrhea patients (%)^*^	3(25)	4(33.3)	3(25)	2(16.7)	0.841^NS^
No. of oligomenorrhea patients (%)^*^	6(22.2)	6(22.2)	7(25.9)	8(29.6)	0.864^NS^
No. of hirsute patients (%)^*^	5(33.3)	5(29.4)	4(23.5)	5(29.4)	0.864^NS^


Data are shown as mean ± SD. Analysis was performed by ANOVA followed by the Tukey’s test for multiple comparisons. * ; Analysis was performed by Pearson Chi-squared test for comparisons, NS; No differences were observed between the mean of variables in the experimental groups compared with placebo, NAC; N-acetyl cysteine, MTF; Metaformin and PLA; Placebo.

**Table 2:** Clinical features before and after treatment in PCOS patients


Parameters		Treatment groups with				
		NAC	MTF	NAC+MTF	PlA	P value

**Weight(Kg)**	Before	72.13±11.9	71.73±8.3	71.26±10.1	71.26±7.2	0.993^NS^
	After	71.53±11.1	71.13±6.7	70.73±9.2	72.6±7.05	0.979^NS^
**Height(cm)**	Before	160.53±5.31	160.46±6.7	160.2±5.2	161.8±7	0.887^NS^
	After	160.53±5.31	160.46±6.7	160.2±5.2	161.8±7	0.877^NS^
**BMI(kg/m^2^)**	Before	27.68±4.5	27.91±3.1	27.78±3.6	26.88±2.3	0.853^NS^
	After	27.06±3.5	26.83±2.3	27.22±3.2	27.16±2.1	0.983^NS^
**Waist size (cm)**	Before	93.9±11	91.13±15.3	90.9±13.2	91.7±15.4	0.931^NS^
	After	90.6±11.1	89.6±13.4	90.5±9.2	92.2±13.9	0.947^NS^
**Hip size (cm)**	Before	108.3±11.3	107.4±13.5	106.4±13.1	108.4±13.4	0.970^NS^
	After	107.6±10.2	105.4±13.1	105.8±10.7	108.8±13.8	0.874^NS^
**WHR**	Before	0.86±0.04	0.84±0.04	0.84±0.04	0.83±0.05	0.366^NS^
	After	0.83±0.04	0.82±0.04	0.83±0.04	0.84±0.06	0.819^NS^


### Biochemical characteristics

Serum levels of LH, total testosterone, cholesterol and triglyceride, insulin and leptin significantly reduced in the MTF and NAC groups compared to the placebo (p<0.05). But levels of LH, total testosterone, cholesterol and triglyceride had no significant reduction in the MTF+NAC groups compared to the placebo (p>0.05). The serum levels of insulin and leptin reduced significantly after treatment in the MTF+NAC group compared to the placebo (p<0.01). Unlike the MTF+NAC and NAC groups, the level of MDA in the MTF group was not significantly decreased compared to placebo group (p>0.05). Levels of FBS, FSH, PRL, E2, DHEA-S, LDL, HDL, VLDL and AMH ratio were not significantly different in treatment groups and to those of the placebo group, data are presented in table 3.

**Table 3:** Biochemical and hormonal parameters before and after treatment in serum of PCOS patients


Parameters		Treatment groups with				
		NAC	MTF	NAC+MTF	PlA	P value^a^

**Fasting insulin (mIU/L)**	Before	17.5±1.61	17.45±1.97	17.88±1.91	18.04±2.25	0.810^NS^
	After	16.07±1.8	16.05±2.4	16.42±2	18.61±2.5	0.005
**FBS (mg/dl)**	Before	97.6±10.5	98.6±8.5	94.7±13.1	95.7±15.2	0.806^NS^
	After	94.26±16.4	93±10.9	93.93±12.8	98.26±13.7	0.727^NS^
**LH (mIU/ml)**	Before	11.51±2.47	10.68±1.88	10.47±1.53	10.77±2.41	0.566^NS^
	After	8.7±2.4^c^	9.5±2.8^b^	10.03±3.4 ^NS^	12.53±3.3	0.007
**FSH (mIU/ml)**	Before	5.3±1.65	4.9±1.58	5.25±0.97	5.46±0.96	0.709^NS^
	After	6.33±1.74	6.1±2.3	6.04±1.64	5.9±1.58	0.962^NS^
**Total testostrone (ng/ml)**	Before	1.11±0.54	1.1±0.48	1.01±0.4	1.19±0.48	0.799^NS^
	After	0.8±0.3^c^	0.76±0.26^b^	0.95±0.34^NS^	1.21±0.42	0.002
**E2(pg/ml)**	Before	69.06±14.6	69.22±13.6	64.59±14.4	70.22±16.6	0.734^NS^
	After	72.22±12.3	72.38±8.2	73.5±8	79.5±8.7	0.128^NS^
**PRL(ng/ml)**	Before	13.53±4.4	13.59±4	14.40±4.6	14.9±5.2	0.817^NS^
	After	15±4.2	16.1±4.6	15.4±4.7	16.75±5.4	0.764^NS^
**DHEA-S(µg/ml)**	Before	2.22±0.93	2.35±1.08	2.14±1.05	2.31±0.97	0.941^NS^
	After	1.85±0.92	1.76±0.76	1.91±0.76	2.34±0.96	0.267^NS^
**Cholesterol (mg/dl)**	Before	184.8±15.1	187.5±21.6	188.5±15.9	188.3±17.1	0.937^NS^
	After	168.2±19.6^c^	169±19.3^b^	173.5±17.5^NS^	190.3±14.8	0.004
**Triglyceride (mg/dl)**	Before	154.2±55.6	150.2±51.1	155.1±45.8	169.1±34.9	0.717^NS^
	After	136.8±24.9^c^	139.2±24.8^b^	145.1±38.9^NS^	171.2±39.1	0.021
**LDL(mg/dl)**	Before	97.9±16.4	95.1±22.3	94.5±18.5	92.4±18.7	0.887^NS^
	After	88.9±21.9	87±22.7	88.8±21.3	93.1±22.7	0.895^NS^
**HDL(mg/dl)**	Before	45.9±6.9	43.6±9	43.3±7.3	42.4±6.9	0.627^NS^
	After	48.1±8.6	49.1±9.2	47±7.5	42.06±6.2	0.086^NS^
**VLDL(mg/dl)**	Before	34.7±12.1	31.3±12.5	34.2±10.1	35.2±8.9	0.743^NS^
	After	31±9.6	30.9±11	32.07±10	39.9±8.3	0.051^NS^
**AMH (ng/ml)**	Before	5.79±1.6	5.47±2.2	5.8±1.5	6.5±1.47	0.429^NS^
	After	5.55±1.6	5.37±1.2	5.61±1.3	6.74±1.45	0.051^NS^
**Leptin (ng/ml)**	Before	23.65±3.3	24.39±3.5	23.82±3.7	24.15±3.7	0.942^NS^
	After	20.05±2.5	19.73±2.1	21.42±2.4	24.48±3.1	0.0001
**MDA (µM)**	Before	7.64±2.64	6.98±2.42	7.8±2.51	7.75±2.05	0.775^NS^
	After	5.76±2.16^c^	6.57±1.98^NS^	6.03±1.9^d^	8.08±2.26	0.017


Data are shown as mean ± SD. Analysis was performed by ANOVA followed by the Tukey’s test for multiple comparisons. Significant differences for the comparison between treatments are in bold type. a; Differences were observed between in the experimental groups compared with placebo,b; MET group compared with placebo 6 weeks after treatment.(p < 0.05) , c; NAC group compared with placebo 6 weeks after treatment.(p < 0.05) and d;. MTF+NAC group compared with placebo 6 weeks after treatment (p< 0.05). NS; No differences were observed between the mean values of variables in the experimental groups compared with placebo (p > 0.05).

Our results showed a significant positive correlation between insulin with BMI (r=0.553, p=0.0001), LH (r=0.371, p=0.004), E2 (r=0.699, p=0.0001) and TT (r=0.544, p=0.0001) before drug administration. Although the serum levels of leptin showed a significant positive correlation with BMI (r =0.379, p=0.003), insulin (r=0.592, p=0.0001), E2 (r=0.547, p=0.0001) and TT (r =0.549, p=0.0001), but no significant correlation was found between leptin concentrations with LH (r=0.168, p=0.201) and FSH (r=-0.143, p=0.276) before drug treatment. However, there was only significant positive correlation between leptin and insulin (r=0.562, p=0.0001) after drug administration. Our results also revealed that MDA levels showed a significant positive correlation with insulin (r=0.307, p=0.017) and TT (r=0.332, p=0.009) before drug administration, data are demonstrated in table 4. Moreover, there was only significant positive correlation between MDA and insulin (r=0.591, p=0.0001) after drug administration (Table 4).

Upon performing multivariate analyses after adjusting for BMI, LH, FSH, E2, TT, insulin and MDA on leptin there was a strong statistically significant relationship between leptin and both insulin (p<0.01) and MDA (p<0.05, Table 5). None of other variables showed statistically significant effect.

Considering the amount of data and length of the manuscript, the data regarding clinical outcomes of ICSI will be presented in a different manuscript.

**Table 4:** Correlations between blood serum variables in the four groups of PCOS patients before and after treatment


Parameters		Insulin	P value	leptin	P value	MDA	P value

**BMI(kg/m^2^)**	Before	0.553	0.0001	0.379	0.003	0.123	0.348
	After	0.008	0.953	-0.062	0.639	-0.139	0.289
**E2(pg/ml)**	Before	0.699	0.0001	0.547	0.0001	0.240	0.065
	After	0.245	0.06	0.112	0.395	0.182	0.164
**LH(mIU/ml)**	Before	0.371	0.004	0.168	0.201	-0.074	0.574
	After	0.190	0.147	0.107	0.415	0.186	0.155
**FSH(mIU/ml)**	Before	-0.173	0.186	-0.143	0.276	-0.125	0.340
	After	0.099	0.452	0.415	-0.055	-0.114	0.388
**TT(ng/ml)**	Before	0.544	0.0001	0.549	0.0001	0.332	0.009
	After	0.081	0.540	0.228	0.08	0.219	0.092
**Insulin(mIU/L)**	Before	-	-	0.592	0.0001	0.307	0.017
	After	-	-	0.562	0.0001	0.591	0.0001


Significant differences for the comparison between treatments are in bold type. MDA; Malondialdehyde, E2; Estradiol, LH; Luteinizing hormone, FHS; Follicle stimulating hormone and TT; Total testostrone.

**Table 5:** Multiple linear regression analysis of the leptin level on insulin and MDA before and after treatment


	Before treatment					After treatment				
	Coefficient	Standard error	P value	95% Coefficient interval		Coefficient	Standard error	P value	95% Coefficient interval	
				lower	upper				lower	upper

**Insulin(mIU/L)**	0.683	0.213	0.002	0.256	1.11	0.500	0.170	0.005	0.159	0.840
**MDA(µM)**	0.323	0.153	0.039	0.017	0.629	0.434	0.183	0.021	0.068	0.799


Significant differences for the comparison between treatments are in bold type. MDA; Malondialdehyde.

## Discussion

PCOS has been a research subject over the past six decades, while it is associated with insulin resistance, hyperandrogenism, obesity, dyslipidemia and infertility (16). The beneficial effects of MTF, an insulin sensitizer (5-7), and NAC (9, 11, 17) on biochemical and clinical aspects of PCOS have been demonstrated by many studies. MTF appears to reduce the output of hepatic glucose, declining insulin concentrations and reducing the androgen production by theca cells (18). On the other hand, NAC is commonly used as a safe drug which increases the cellular levels of reduced glutathione. NAC also influences insulin receptor activity and significantly decreases T levels and free androgen index values (9, 17).

Considering the benefits of MTF and NAC consumption in patients with PCOS, we decided to investigate whether a combination therapy of MTF and NAC during the course of ovarian stimulation during ICSI cycle in patients with PCOS will introduce a better effect in reducing hyperinsulinism and hyperandrogenism and in normalizing other biochemical and endocrine parameters rather than using MTF or NAC alone. We should stress that our study is a pilot study, and there is a lack of literature on combined administration of these drug, especially during course of ovarian stimulation.

Our results showed that 6 weeks of co-treatment with NAC and MTF is significantly effective in reducing the levels of insulin compared with the control. Although this result supports previous studies which have reported a significant change in the fasting insulin levels after the consumption of MTF and NAC compared with the placebo group(17), and this is the first report of the reduced level of insulin following combination therapy. There is also some contrary reports (9, 19, 20) which could probably be due to the differences in patient inclusion criteria, dosing and duration of therapy, and genetic background of individuals.

Concomitant with reduced hyperinsulinaemia, a significant decline in the serum levels of leptin upon treatment with MTF, NAC and MTF+NAC groups was observed compared to the control group. This observation was consistent with background literature on MTF and NAC (21). These observations are in line with previous report on leptin which is considered to play an important role in the pathogenesis of PCOS that influences the LH production (22). Furthermore, in our study, a significant positive correlation was observed between leptin and insulin concentrations before and after treatment, indicating that insulin reduction was responsible for lowering leptin secretion. Moreover, it should be emphasized that when multiple regression analysis was performed, such correlation was found. Other study (23) have also shown that leptin concentrations correlated significantly and positively with insulin in women with PCOS. On the other hand, in a study of Yarali et al. (19), six weeks of treatment with 850 mg MTF twice daily made no significant decrease in serum leptin levels compared to the placebo, which is probably due to the differences in drug doses and protocols of ovulation induction.

Our results revealed a significant reduction in the levels of TT after 6 weeks of treatment with NAC and MTF alone, which is in consistent with previous findings (17). Insulin directly stimulates androgen production from theca-cells by increasing the activity of cytochrome P450-C17a, and it also induces adrenal esteroidogenesis (16). Therefore, MTF lowers the level of serum androgens through direct inhibition of androgen production in human ovarian theca cells and also by improving insulin sensitivity, indirectly (24). On the other hand, NAC decreases the expression of several key enzymes involved in T production such as 17β-hydroxysteroid dehydrogenase (17β-HSD) and 3β-hydroxysteroid dehydrogenase (3β-HSD)(25), leading to a decrease in the levels of T production. However, in contrary to our expectations, MTF and NAC combination did not cause a significant decrease in testosterone levels, probably due to the effects of drug interference which need future investigation. In our study, as well as previous study (22), leptin concentrations correlated significantly and positively with TT in PCOS patients, which likely suggests that a decrease in leptin levels can reduce esteroidogenesis. An abnormal lipid profile is a common finding in PCOS which elevates levels of total cholesterol, triglycerides, and LDL, while decreases levels of HDL (26). In our study, similar to previous study (27), an improved lipid profile was obtained following NAC and MTF administration by means of reduced TG and total cholesterol. These effects were not observed in combination therapy, and we have no suggestion for these observations, but drug co-treatment remains to be explored and needs future validation.

Several studies have pointed out an increase in oxidative stress in PCOS patients which is influenced by the following factors: age, hyperhomocysteinemia, hyperandrogenemia and insulin resistance (28, 29). Leptin has peripheral actions to stimulate vascular inflammation, oxidative stress and cardiovascular disease (30). Leptin via Janus kinases(JNK) pathway [one of the intracellular signaling pathways; mitogen-activated protein (MAP)-kinase] increases the transcription factors activity of NF-kB (nuclear factor-kB) and AP-1(activator protein-1), thereby increases reactive oxygen species (ROS) through induction of cellular oxidation (31). A significant positive relationship between increased levels of MDA, as a marker of lipid-peroxidation and of increased oxidative stress, and the total testosterone and insulin levels in PCOS patients was observed in this study, confirming the existence of increased oxidative stress in patients with PCOS (32). In our study, following treatment, the level of MDA in the MTF+NAC group was not significantly different from the NAC group, but has a significant reduction compared to MTF group. This could be due to the fact that MTF may raise oxidative stress through increased homocysteine levels which is associated with increased generation of superoxide anions and decreased activity of antioxidant enzymes (32). Since NAC, as an antioxidant, inhibits the transcription factors activity of NF-kB and AP-1, it influences the activity of the signaling pathway of MAP-kinase and results in a reduction in the oxidative stress (11-13); therefore, it is possible that NAC can decrease elevated oxidative stress in PCOS patients taking MTF.

There was no significant reduction in the FBS, PRL, E2, DHEA-S, LDL, HDL and VLDL levels after 6 weeks of treatment with NAC and MTF group, which is in agreement with previous findings (19) about MTF+NAC group as compared with the control. However, the reduction of AMH level in the treatment groups, which was close to be significant (p=0.051), is consistent with literature, so it has been suggested that long-term administration is required for a reduction in the raised AMH level observed in PCOS individuals (33), indicating that these drugs can reduced the number of AMH producing follicle which is appropriate for PCOS patient.

## Conclusion

Despite the fact that MTF and NAC have different influence of action (i.e. one as insulin sensitizers and other as an antioxidant), we observed no beneficial effect of these drugs which administered together during course of ovulation induction for ICSI. In contrast to our expectation, the beneficial effect of each drug on reducing LH, TT, cholesterol and TG which was observed when MTF or NAC was administered alone rather than combination therapy. Therefore, we conclude that co-administration of these drugs might not be useful for PCOS patient during course of ovulation stimulation. However, the beneficial effects of co-administration of the two drugs for routine treatment of PCOS patient remain to be evaluated.
